# Basal cytokines profile in metastatic renal cell carcinoma patients treated with subcutaneous IL-2-based therapy compared with that of healthy donors

**DOI:** 10.1186/1479-5876-5-51

**Published:** 2007-10-22

**Authors:** Michele Guida, Addolorata Casamassima, Giulia Monticelli, Michele Quaranta, Giuseppe Colucci

**Affiliations:** 1 Department of Medical Oncology, National Oncology Institute, Bari, Italy; 2 Departments of Experimental Oncology, Laboratory of Immunology, National Oncology Institute, Bari, Italy

## Abstract

**Background and purpose:**

Metastatic renal cell carcinoma (MRCC) has a poor prognosis with a median overall survival of about one year. Since only a minority of patients experienced therapeutic benefit to current treatments, several studies have attempted to identify factors that may have an impact on response and survival. Cytokines play a crucial role in the host's immune response by regulating the development and function of a lot of biological compartments. Nevertheless, available data on basal cytokine levels in MRCC are very few and no clear profile of serum cytokines has been identified yet in these patients population. Thus, determining the levels of cytokines in MRCC could not only help in understanding the biological mechanisms of the tumor growth, but also in evaluating if different cytokine profiles are correlated with particular clinical behaviors.

**Materials and methods:**

In 144 healthy donors and 55 MRCC treated with subcutaneous IL-2-based regimens, we analysed a panel of basal cytokines particularly involved in the neoplastic progression (IL-1beta, IL-6, IL-8, IL-10, IL-12, alpha-TNF) and C-reactive protein (CRP) in order to compare their levels in the two groups, and to verify their impact on patient response and survival.

We first compared cytokines levels in patients population and healthy donors. Than, in definite patients group, univariate and multivariate analyses were performed to evaluate the correlation existing between each factor considered and clinical outcomes. For these analyses, baseline values were included as dichotomous variables using the median values (above and below) of control group.

**Results:**

In general, higher levels of cytokines were found in patients with respect to those of healthy donors, both in term of percentage of undetectable levels or median values. The impact on response was insignificant, except for higher levels of CRP that were strongly correlated with a worse response (p < 0.001). Within the patients groups, a worse survival was associated with higher values of CRP (8 vs 31 months, p = 0.0000), IL-6 (9 vs 25 months, p = 0.0295), and IL-8 (9 vs 17 months, p = 0.0371). Conversely, higher levels of IL-12 were associated with a better survival (25 vs 15 months, months p = 0.0882). A correlation was found between CRP and IL-6 (p = 0.009) and between CRP and IL-10 (p = 0.038). After multivariate analysis only CRP (p = 0.0035) and IL-12 (p = 0.0371) maintained an independent impact on survival, while IL-6 showed a borderline value (p = 0.0792).

**Conclusion:**

Higher cytokines levels characterize patients population with respect to healthy donors. Moreover, higher basal level of some immunosuppressive cytokines (CRP, IL-6, IL-8) result correlated with a poorer survival, whereas higher levels of IL-12, a cytokine with a potent antineoplastic activity, was associated with a better survival. A wider sample of patients is needed to better clarify if our findings are intrinsically related to patients population or if they are simply an epiphenomenon of disease progression.

## Background

Renal cell carcinoma (RCC) is a chemoresistant disease and also immunotherapy-based regimens are considered of a modest efficacy [1]. In fact, metastatic RCC (MRCC) has a poor prognosis with a median overall survival of about one year until few years ago [2]. Recombinant Interleukin-2 (IL-2) and interferon alpha (α-IFN) were the most widely used cytokines in MRCC inducing objective response rates from 15% to 20% as monotherapy or as combined regimens [3,4]. The efficacy of adding chemotherapy to immunotherapy in MRCC remains questionable. In our experience, a very high percentage of response was obtained with a bi-weekly regimen including subcutaneous IL-2, Vinorelbine and Gemcitabine [5]. Recently, some multi-target oriented drugs have shown an impressive activity in MRCC with a high percentage of partial response and/or stable disease with a significant impact on survival [6,7]. Nevertheless, immunotherapy remains another important therapeutic option for these patients thanks to its curative potential in some patients and its capability to obtain very durable responses as demonstrated by long-term follow-up. Therefore, the increasing therapeutic options for RCC should be seen not as a competition among the different treatments, but as an expanding armamentarium available for these patients.

Since only a part of patients experienced therapeutic benefit to the current treatments, several studies have attempted to identify factors that may have an impact on response and survival in MRCC patients. Some previously published studies identified a number of clinical and standard bio-humoral parameters correlated with a poorer survival and predictive of rapid progression [8-11], but at present data regarding basal cytokines profile in MRCC are very few.

Cytokines regulate cellular immune interactions and are produced by lymphocytes, monocytes, macrophages, and, some cytokines, also by fibroblasts, neutrophils, endothelial cells, or mast cells [12,13]. They play a crucial role in the host's immune response by regulating the development and function of a lot of biological compartments as the immunological and angiogenic ones [14]. Normally, cytokines are most commonly assessed at the macro-environmental levels by measuring serum and plasma levels or levels in the supernatant of in *vitro*-stimulated blood cells; nevertheless, these data do not reflect their real work in an *in vivo *micro-environmental contest [13]. Moreover, a lot of sub-clinical situations and chronic co-morbidity could influence cytokines profile in patients with metastatic neoplasms. Since cytokines are released in a large number of inflammatory, non-neoplastic conditions, when detected in the serum of cancer patients, they cannot be used as specific markers of neoplastic disease, but rather as markers of the overall serological pattern of concomitant diseases.

No clear profile of serum cytokine has been identified yet in the patients with metastatic tumours, and it does not exit a clear cytokines cut-off between patients and health subjects. Finally, it is not clear what are the levels of cytokines able to discriminate normal and pathological situation and what is the significance of cytokines modifications during the tumor progression or during some biological therapy such as IL-2 therapy. Finally, it is not clear if different cytokine profiles are correlated with particular clinical behaviors.

Our and other groups have recently demonstrated that C-Reactive Protein (CRP) has a strong negative impact of response and survival in MRCC [15]. In our previous experience we analysed in the same population of the present study, a list of clinical and serum parameters in order to verify their prognostic and predictive significance. In the univariate analysis, we found that a good PS (P = 0.0000), prior nephrectomy (P = 0.0001), disease free interval (DFI) longer than 12 months (P = 0.0003), bone disease site (P = 0.0013), low number of metastatic site (P = 0.0449), normal albumin (P = 0.0001), low/normal fibrinogen (P = 0.0140), low/normal LDH (P = 0.0430) and low/normal CRP (P = 0.0000) were related to a better survival; nevertheless in the multivariate analysis, only CRP (P = 0.002) and DFI (P = 0.0497) were found to have an independent role on survival. For this reason we also included in the analysis of the present study these two significant parameters.

We analysed in 144 normal donors and in 55 patients affected by MRCC treated with different regimens including low dose subcutaneous IL-2, the basal levels of a panel of cytokines particularly involved in the neoplastic pregression processes (IL-1 beta, IL-6, IL-8, IL-10, IL-12, α-TNF) and CRP in order to compare their profiles in the two groups, and to verify their impact on patient response and survival.

## Methods

### Patients and treatment

We analysed a total of 55 patients with MRCC of which we had available serum. All patients were treated at the Oncology Institute of Bari, Italy in three phase II studies approved by the local ethics and carried out in the past 5 years. An histological proven diagnosis of RCC and an adequate Eastern Cooperative Oncology Group (ECOG) performance status (0–2) were also requested. Tumour response was defined according to the WHO criteria. Data from the three studies were pooled and than analysed.

Treatment consisted of low dose subcutaneous IL-2 alone (15 patients), or IL-2 plus chemotherapy including Vinblastine (20 patients), or Gemcitabine plus Vinorelbine (20 patients). The main characteristics of patients are shown in Table 1.

**Table 1:** Patients characteristics

Characteristics	
Median ECOG PS (range; PS 0/1/2)	1 (0–2; 10/40/5)
Median age (range)	62 (27–82)
Sex	
men (%)	35 (64%)
woman (%)	20 (36%)
Disease site (%)	
Brain	2 (4%)
Bone	11 (20%)
Liver/viscera	23 (43%)
Soft tissue/lung	18 (33%)
Pts metastatic sites (%)	
1	16 (30%)
> 1	39 (70%)
Median Disease Free Interval (range)	4 (0–240)
Nephrectomy (%)	
Yes	50 (91%)
No	5 (9%)

Clinical results, irrespective of the regimen used, included 21% of complete plus partial response (CR + PR), 29% of stable disease (SD) and 50% of progressive disease (PD). Median overall survival for all patients was 9 months (1–40 months), 15 months for CR + PR patients (4–40 months), 14 months for SD patients (2–36 months) and 6 months for PD patients (1–35 months), respectively.

### Sample analysis

We analysed a panel of cytokines (IL-1b, IL-6, IL-8, IL-10, IL-12 and TNF-α) both in patients population and healthy donors constituted by individuals without inflammatory and chronic diseases. This group was comparable with the patients group regarding age, gender and race distribution. Moreover, in the patient group, we also included CRP levels previously identified as a strong negative factor of response and survival in MRCC [15].

Serum samples were obtained from patients on the day prior to the beginning of treatment and stored at -80°C. Serum samples were also obtained from a control population of 144 healthy donors with comparable sex and age distribution. Each sample was tested in a double aliquot for each different cytokine. Informed consent was obtained from all patients and control subjects.

Rate nephelometry was employed in the quantitative determination of CRP (Beckman Coulter); normal range considered has been < 0.8 mg/dl, IL-1 beta, IL-6, IL-8, IL-10, IL-12, and alpha TNF serum levels were measured by a powerful multiplexed assay combined with flow cytometry using commercially available kits BD™ Human Inflammation Cytometric Bead Array (CBA) and according to the kit procedure (BD Biosciences Immunocytometry Systems and BD Bioscience Pharmigen). The inflammation CBA assay, simultaneously quantifying IL-1b, IL-6, IL-8, IL-10, IL-12p70, and TNF-a in a single sample, used 25 μl of serum sample. Incubation time was 6 h, CBA beads were analyzed with a FACS Can flow cytometer (BD Biosciences) and analyzed with the proprietary software. The detection limit of kit was 0 pg/ml for all cytokines.

We first evaluated cytokines levels both in patients population and healthy donors. Results were expressed as percentage of detectable values and as median values in both groups. Than, we divided our patients into two different groups using the median values obtained from the control group (above and below the median value of healthy donors). The response rate and overall survival were analyzed in the two groups of patients.

### Statistical analysis

Data were analyzed using the SPSS software package (SPSS, Inc., Chicago, IL, USA). Statistical significance was defined at p < 0.05 for univariate and multivariate analyses. Standard curves were generated for each assay and experimental values were computed by using a regression analysis. Survival curves were traced according to Kaplan-Maier and differences analyzed with *log-rank test *(level of significance < 0.05. The t-test was used to compare the basal levels of bio-humoral parameters (normal range versus abnormal range) with respect to the clinical response. As regards the response analysis, 95% confidence interval (95% CI) of response rate was calculated, and comparison between groups was assessed using chi-square's test. We used univariate analysis to evaluate the relation between survival and response to treatment with respect to the baseline cytokines and CRP levels. The cytokines and CRP were included in the model as a dichotomous variables in two categories: above and below the median values of healthy donors for cytokines and inferior or superior to normal value for CRP. We used the median rather than the mean of variables considered in statistical tests because of their not gaussian distribution.

All the parameters resulted significant in the univariate analysis were considered for the multivariate analysis. The Cox proportional hazards model was used in multivariate analyses to study the prognostic impact of the different variables on survival. From an initial model, using a backward selection procedure, a final parsimonious model was obtained. Results of these analyses were reported in risk ratios of dying with 95% confidence intervals (CIs) [16]. We also performed a no-parametric test of the median to compare median normal value of cytokines of healthy donors with respect to those of the patients.

## Results

We analysed the cytokines levels of 144 healthy donors and of 55 patients with MRCC. When we compared the cytokines levels of healthy donors with those of patients, we found undetectable levels of IL-1b in the 0.1% of healthy donors compared to 0% of patients (p = 0.131); IL-6 was undetectable in the 27% of healthy donors compared to 24% of patients (p = 0.858); IL-8 was undetectable in the 41% of healthy donors compared to 28% of patients (p = 1); IL-10 was undetectable in the 56% healthy donors compared to 19% of patients (p < 0.001); IL-12 was undetectable in the 32% of healthy donors compared to 0.1% of patients (p = 0.003); TNF-α was undetectable in the 64% of healthy donors compared to 19% of patients (p < 0.001).

**Figure 1:**
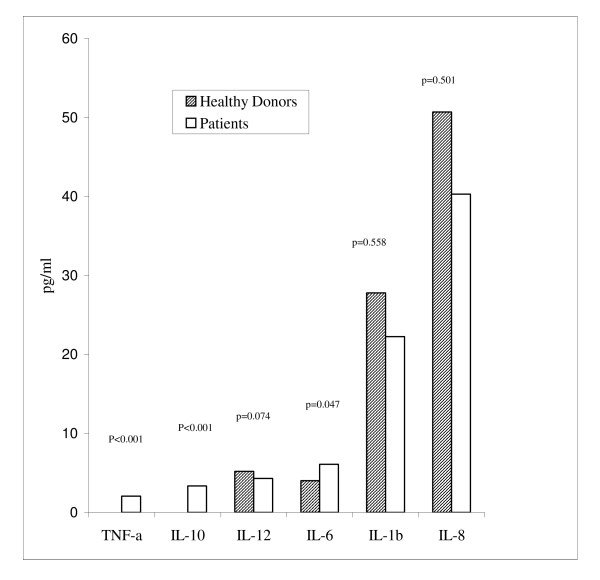
No-parametric test comparing the median levels of cytokines in healthy donors and patients.

We also expressed our data as median values in both healthy donors and patients group. Data regarding the donors are as follow: IL1beta = 27.8 pg/ml (range, 0 to 514.1 pg/ml), IL6 = 4 pg/ml (range, 0 to 85.7 pg/ml), IL8 = 50.7 pg/ml (range, 0 to 570.1 pg/ml), IL10 = 0 pg/ml (range, 0 to 21.3 pg/ml), IL12 = 5.2 pg/ml (range, 0 to 126.7 pg/ml), alpha TNF = 0 pg/ml (range, 0 to 45.8 pg/ml). In the patient group, the median values were: IL1beta = 22.25 pg/ml (range, 0 to 779.8 pg/ml), IL6 = 6.1 pg/ml (range, 0 to 136.3 pg/ml), IL8 = 40.3 pg/ml (range, 8.1 to 445.3 pg/ml), IL10 = 3.35 pg/ml (range, 0 to 49 pg/ml), IL12 = 4.3 pg/ml (range, 0 to 435.15 pg/ml), alpha TNF-α = 2.05 pg/ml (range, 0 to 15.7 pg/ml). When we compared the median values of cytokines of the 144 healthy donors with those of the 55 patients, we found a significant difference just for TNF-α (p < 0.001), IL-10 (p < 0.001) and IL-6 (p = 0.047); IL-12 showed a difference near the statistical significance (p = 0.074) (Fig. 1).

**Figure 2:**
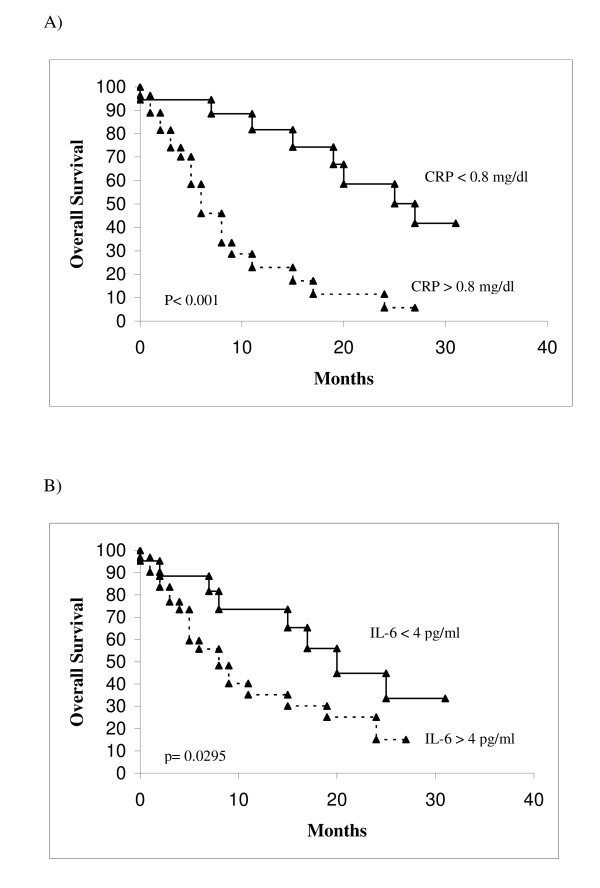
Overall survival in patients according to significant univariate analysis parameters. A, CRP < 0.8 VS > 0.8 mg/dl. B, IL-6 < 4 vs > 4 pg/ml.

**Figure 3:**
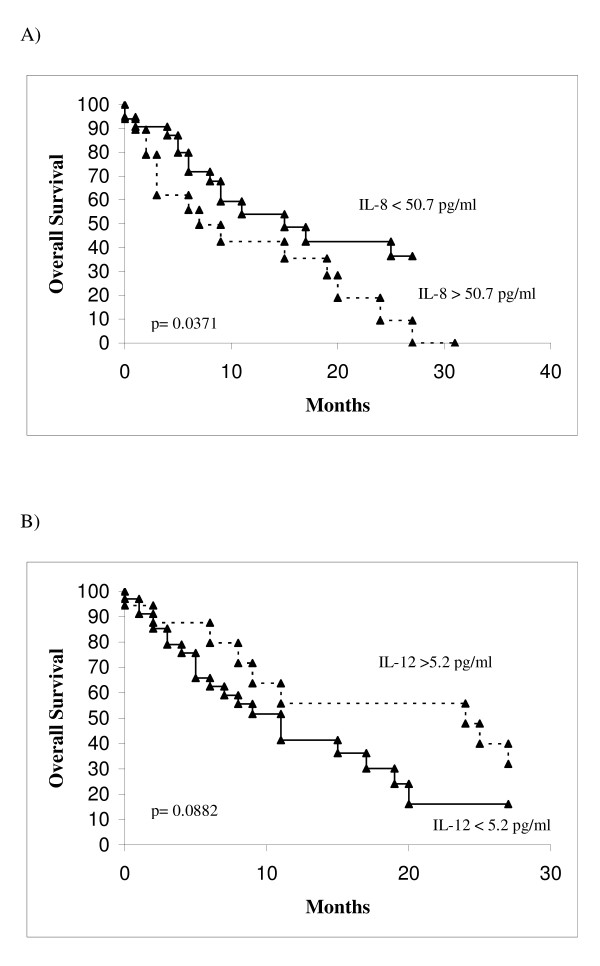
Overall survival in patients according to significant univariate analysis parameters. A, IL-8 < 50.7 vs > 50.7 pg/ml. B, IL-12 < 5.2 vs > 5.2 pg/ml.

Table 2 summarizes the correlation between the cytokines and survival. In univariate analysis, higher values of IL-6 were found to correlate with a worse survival with respect to lower values: 9 months and 25 months respectively (p = 0.0295). Also the CRP and IL-8 had the same capability to discriminate patients with a different survival as IL-6: higher levels of CRP were correlated with a poorer survival (8 months with respect to 31 for lower levels) (p < 0.0001); higher levels of IL-8 were also correlated with a poorer survival (9 months with respect to 17 months for lower levels) (p = 0.0371). Regarding IL-12, a better survival was correlated with higher levels of this cytokine (25 months) with respect to lower levels (15 months), but the difference was not significant months (p = 0.0882). Conversely, the different levels of IL-1b, IL-10 and TNF-α are not able to discriminate patients with different survival. Figures 2 and 3 summarize the survival curves of patients according to significant cytokines (IL-6, IL-8 and IL-12) and CRP.

**Table 2:** Results of the univariate analysis testing the panel of cytokines and CRP

Parameters	% Pts	Median Survival (CI) (mos)	p Value (log rank)
IL-Iβ:			
< 27,8 pg/ml	57	11 (5.05–16.95)	0.1761
> 27,8 pg/ml	43	24 (14.20–33.80)	
IL-6:			
< 4 pg/ml	40	25 (11.82–38.18)	0.0295
> 4 pg/ml	60	9 (5.44–12.56)	
IL-8:			
< 50,7 pg/ml	62	17 (0.00–34.06)	0.0370
> 50,7 pg/ml	38	9 (0.00–23.16)	
IL-10:			
< 0 pg/ml	19	19 (1.82–36.18)	0.3954
> 0 pg/ml	81	15 (7.04–22.96)	
IL-12:			
< 5,2 pg/ml	64	15 (6.93–23.07)	0.0882
> 5,2 pg/ml	36	25 (19.87–30.13)	
TNF-α:			
< 0 pg/ml	19	7 (0.80–13.20)	0.1159
> 0 pg/ml	81	20 (10.56–29.44)	
CRP:			
< 0,8 mg/dl	46	31 (21.29–40.71)	< 0.0001
> 0,8 mg/dl	54	8 (5.68–10.32)	

Abbreviations: CRP, C-reactive protein; IL, interleukin; TNF, tumor necrosis factor

The impact of baseline cytokines on response was insignificant. Only higher levels of CRP were strongly correlated with a worse response (p < 0.001). The majority of progressive patients showed high value of CRP (73.1%; 95% CI, 56% to 89%) compared to patients with PR+CR/SD (26.9% with value above the normal) (p = 0.003). Moreover, when we carried out a chi-square test on associations between cut-off variables, we just found an association between CRP and IL-6 (p = 0.009), and CRP and IL-10 (p = 0.038).

We also carried out a multivariate analysis on the 55 patients considering the panel of cytokines and CRP, using a backward selection procedure. At the final parsimonious model, only CRP (p = 0.0035), and IL-12 (p = 0.0371) were found to have independent impact on survival; in addition IL-6 showed a borderline value (p = 0.0792) (Table 3).

**Table 3:** Final multivariate model

**Variables**	**HR**	**95% CI**	p
IL12 (< 5.2 vs > 5.2 g/ml)	0.3525	0.1323–0,9394	0.0371
IL6 (< 4 vs > 4 pg/ml)	2.3115	0.9070–5.8911	0.0792
IL10 (< 0 vs > 0 pg/ml)	3.0490	0.7458–12.4647	0.1207
CRP (< 0.8 vs > 0.8 mg/dl)	4.0361	1.5801–10.3099	0.0035

## Discussion

Immunotherapy remains one of the therapeutic options for patients with MRCC. In particular, IL-2 have demonstrated to obtain very durable responses and stable disease in some patients. Nevertheless, the antineoplastic mechanisms of IL-2 and its effects on peripheral blood mononuclear cells and T cell subsets are only partially known [2,3,17,18].

The evaluation of cytokines in the serum of neoplastic patients and in normal subjects is arduous because of a series of concomitant sub-clinical situations and acute or chronic co-morbidities. Moreover, it does not exist a clear cytokine cut-off between patients and healthy subjects, and it is also unclear what are the levels of cytokines able to discriminate normal and pathological conditions [14]. Available data on basal cytokine levels in MRCC are very few. No clear profile of serum cytokines has been identified yet in these patients population and even less is known about cytokine behaviour during treatment [18].

We evaluated a panel of basal cytokines more commonly involved in the tumor control and progression (IL-1 beta, IL-6, IL-8, IL-10, IL-12, TNF-α) both in 144 healthy donors and in 55 patients affected by MRCC treated with IL-2 based regimens. Because we and other group have recently demonstrated that C-Reactive Protein (CRP) has a strong negative impact of response and survival in MRCC [15], we also included the CRP levels among the parameters analysed in the present study.

It is known that a possible imbalance in the type1 (IL-12 and Interferon-γ), and type 2 cytokine (IL-6, IL-10) pattern has been postulated in neoplastic patients [18]. In particular, IL-6 is a multipotent cytokine exerting numerous biological activity. It regulates the proliferation and differentiation of immunocompetent cells including T- and B-lymphocytes, NK cells, normal hematopoietic progenitors, epithelial and neural cells. IL-6 is also produced by several epithelial cancer cell lines and it may act as possible growth factors in advanced melanoma. IL-6 is also able to inhibits cell-cell adhesion and to promote spreading of cancer cells and to inhibit T-cell proliferative response. IL-6 is also a potent pro-inflammatory cytokine. It acts as an endogenous pyrogen and induces the expression of the acute phase protein genes including the CRP gene. Finally, IL-6 blocks apoptosis induced by p53, TGFβ, and several chemotherapeutic agents. IL-6 is usually co-expressed together to IL-10, another potent suppressor of immune functions including antigen presentation, cytokine production, macrophage activation, and antigen-specific T-cell activation [18]. IL-10 downregulates class II MHC expression (immunosuppressive activity) and inhibits the production of proinflammatory cytokines by monocytes. Higher levels of IL-10 have been found in metastitic melanoma patients with respect to healthy donors. Nevertheless, the role of both these cytokine is complex and not completely clear yet. In experimental model both IL-6 and IL-10 exert antitumor effects in mice and enhance the major histocompatibility-restricted and non-restricted cytotoxic activity. IL-8 and IL-12 probably play a less relevant role in neoplastic diseases. IL-8 is able to induces tumoral cells migration and inhibits the lymphocytes tumor infiltration. Moreover, its serological levels correlate with the tumor load. IL-12 induces a Th1-type response and tumor rejection. It is also able to inhibit angiogenesis induced by tumor cell lines [12-14,18]. CRP seems mainly to reflect IL-6 secretion. Moreover, elevated pretreatment values are frequent in advanced cancer patients with poor prognostic characteristics, such as cachexia. Finally, pretreatment high levels of CRP predict both the lack of IL-2 activity on tumour objective response and its efficacy on overall survival in metastatic cancer patients [15].

Our results suggest that cytokine profile greatly differs between patient population and healthy control. We found a higher percentage of individuals with undetectable levels of cytokines in healthy donors with respect to patients (TNF-α, p < 0.001; IL-10, p < 0.001; IL-12, p = 0.003). When we analysed our data as median values, in patient population we found higher values of TNF-α (p < 0.001), IL-10 (p < 0.001) and IL-6 (p = 0.047) and lower values of IL-12 (p = 0.074), IL-1β (p = 0.558) and IL-8 (p = 0.501) compared with healthy donors (Fig. 1). No correlation was found between the baseline cytokines and response, except for CRP which was inversely correlated with a better response. Regarding data on survival, higher levels of IL-6 (p = 0.0295) and IL-8 (p = 0.0371) and lower levels of IL-12 (p = 0.0882) was associated with a worse survival. Nevertheless, in a multivariate analysis only IL-12 (p = 0.0371; HR 0.3) and CRP (p = 0.0035; HR 4.0) had an independent impact on survival, while IL-6 showing a borderline value (p = 0.0792; HR 2.3).

Moreover, in this study we confirmed that both high levels of CRP and IL-6 are correlated with a poorer survival in MRCC patients (p < 0.001 for CRP and p = 0.0295 for IL-6) with an independent role (p = 0.0035 for CRP and p = 0.0792 for IL-6) and a high grade of association (p = 0.009).

Our findings suggest that probably the pattern of cytokines does not reflect just an inflammatory status, but it is indicative of a particular biological pattern of advanced neoplastic disease. In fact, the patient with a worse survival showed not only higher levels of IL-10, IL-6 and TNF-α, which are notoriously involved in disease progression and in immunosoppressive mechanisms, but also lower levels of IL-12 which has an antineoplastic potential [13,14,18]. These observations suggest that a degree of immunological imbalance exists in the patient group. Nevertheless, because of the small number of sample it is arduous to interpret these results and to draw definitive conclusions. A wider sample of patients is needed to better clarify if our findings are intrinsically related to the patients population or if they are simply an epiphenomenon of disease progression.

In conclusion, we observed that in addition to CRP, also high level of immunosuppressive cytokines such as IL-6 and IL-8, and low level of IL-12, a cytokine with a potent antineoplastic activity, correlated with a poorer survival in MRCC patients. These findings may help to better define a baseline cytokine pattern for this setting of patients.
